# Maternal COVID-19 vaccination status and association with neonatal congenital anomalies

**DOI:** 10.3389/fped.2024.1355502

**Published:** 2024-04-19

**Authors:** Janelle Santos, Megan Miller, Megan E. Branda, Ramila A. Mehta, Regan N. Theiler

**Affiliations:** ^1^ Obstetrics and Gynecology, Mayo Clinic, Rochester, MN, United States; ^2^ Quantitative Health Sciences, Mayo Clinic, Rochester, MN, United States

**Keywords:** neonatal, abnormalities, birth defects, neonatal outcomes, COVID-19, pregnancy, vaccination

## Abstract

**Introduction:**

Despite recommendations for COVID-19 vaccination in pregnant people, the effect of vaccination on neonatal outcomes remains unknown. We sought to determine the association between COVID-19 vaccination status in pregnancy and presence of neonatally diagnosed congenital anomalies.

**Methods:**

A comprehensive vaccine registry was combined with a delivery database to create a cohort including all patients aged 16–55 years with a delivery event between December 10, 2020 and December 31, 2021 at a hospital within the Mayo Clinic Health System. Pregnancy and neonatal outcomes were analyzed in relation to vaccination status and timing, including a composite measure of congenital anomalies diagnosed in neonatal life. Comparisons between cohorts were conducted using chi-square test for categorical and Kruskal–Wallis test for continuous variables. A multivariable logistic regression was modeled to assess the association with congenital anomalies.

**Results:**

5,096 mother-infant pairs were analyzed. A total of 1,158 were vaccinated, with 314 vaccinated in the first trimester. COVID-19 vaccination status, including vaccination during the first trimester of pregnancy, was not associated with an increased risk of composite congenital anomalies. When further examining congenital anomalies by organ system, we did demonstrate a significant difference in eye, ear, face, neck anomalies between vaccinated and not vaccinated groups (Table 3, Not vaccinated = 2.3%, Vaccinated = 3.3%, *p*-value 0.04) however we did not demonstrate this difference between the 1st trimester and not vaccinated groups (Not vaccinated = 2.3%, 1st Trimester = 2.5%, *p*-value 0.77). No differences were found between not vaccinated, vaccinated, or 1st trimester vaccinated groups for any other organ systems. There were no differences in birthweight by gestational age, APGAR scores, incidence of NICU admission, or living status of the neonate by vaccination status.

**Conclusion:**

We add additional information regarding the safety of COVID-19 vaccination status and timing as it pertains to neonatal composite congenital anomalies, with no association demonstrated. Our findings agree with prior literature that COVID-19 vaccination is not associated with adverse pregnancy outcomes or small for gestational age neonates. Further research is needed to elucidate the association between COVID-19 vaccination and eye, ear, face, neck, anomalies.

## Introduction

SARS-COV-2 infection has been associated with increased risks in the pregnant compared to non-pregnant populations. Need for ICU admission, mechanical ventilation, and extracorporeal membrane oxygenation (ECMO) have been shown to be significantly higher among pregnant people with COVID-19 disease (1, 2). Pregnant people with SARS-COV-2 infection also have increased mortality compared to uninfected pregnant people (2, 3). In addition, SARS-COV-2 infection in pregnancy has been associated with adverse pregnancy outcomes including preterm delivery, stillbirth, and increased risk of maternal mortality and morbidity from obstetric complications such as hypertensive disorders of pregnancy and secondary infections (3–7).

In April 2021, the Centers for Disease Control and Prevention (CDC) announced that pregnant people who were eligible for the COVID-19 vaccine should receive it after data from 90,000 participants in vaccine safety registries did not identify any safety concerns in pregnant participants or their offspring (8, 9). Despite reassuring evidence and recommendations for vaccination, two global meta-analyses demonstrated only 49%–54% of pregnant women would be accepting of vaccination (10, 11). There is currently no guidance for vaccine administration at any particular gestational age, and many pregnant people choose to vaccinate in the second or third trimesters to avoid theoretical concerns surrounding the possible effects of vaccination on organogenesis (12). We report on the association between COVID-19 vaccination timing in pregnancy and congenital anomalies as diagnosed in neonatal life.

## Materials and methods

### Study design

Patient information was collected using a comprehensive vaccine registry that was linked to Mayo Clinic as well as the Mayo Clinic Health System delivery registry. The Mayo Clinic Health System is as system of community-based medical facilities owned by Mayo Clinic. The vaccine registry captured COVID-19 vaccine administrations, manufacturers, and patients, as well as identifying information from Mayo Clinic vaccination sites and other sites across the states of Minnesota and Wisconsin. Our delivery registry data are directly derived from elements in the electronic medical record and were used in a previous study (13). All fields were validated manually during development. The creation of the registries and subsequent analysis were performed under approval by the Mayo Clinic Institutional Review Board.

This was a retrospective cohort study including all patients aged 16–55 years with a delivery event between December 10, 2020 and December 31, 2021 at hospitals within an integrated healthcare system. To be included, pregnancy must have achieved at least 20 weeks gestation at time of delivery. Gestational age was established using American College of Obstetrics and Gynecology (ACOG) criteria (14). In accordance with Minnesota law, patients who opted out of using their medical records for research were excluded from the study. Corresponding infants were included if the infant had research authorization as well.

To assess vaccination status and pregnancy outcomes, vaccinated individuals were defined as those receiving any dosage or formulation of the COVID-19 vaccine from 30 days prior to pregnancy onset, defined as 30 days prior to day 1 of pregnancy by ACOG dating, until delivery. Dates of each dose were captured as well as the timing in relation to the pregnancy (pre-pregnancy, 1st, 2nd, 3rd trimester). Vaccination status was categorized as none, at least one dose during the first trimester (1st Tri) and all other vaccinations (Other), which may have occurred pre-pregnancy or during pregnancy as long as no doses occurred during the first trimester (Supplementary Appendix A). Timing of vaccination was determined via electronic health record documentation. Most vaccinations were mRNA vaccines manufactured by either Pfizer or Moderna (Supplementary Appendix B). COVID-19 status indicates the presence of infection during the pregnancy, regardless of temporal relation to the vaccine, defined as a positive SARS-COV-2 result via reverse-transcription-polymerase chain reaction test documented in the medical record between day 1 of pregnancy and delivery.

### Outcomes

The primary outcome of congenital anomalies is a composite outcome calculated as the summation of infants with one or more congenital anomaly diagnosis codes (Supplementary Table S1). Our congenital anomaly list most closely mirrors that used by the Metropolitan Atlanta Congenital Defects Program (MACDP), the reference used by the CDC (15). All anomalies were diagnosed in neonatal life, as opposed to prenatal ultrasound. We used ICD-10 diagnostic codes to identify anomalies which were then verified by manual medical record review of neonatal inpatient and outpatient records. All infants (living, stillbirth, neonatal demise, and therapeutic abortion) were examined for congenital anomalies. If infants had multiple anomalies, they were only counted once in the congenital anomaly composite. For each anomaly type (chromosomal, musculoskeletal, urinary, genital, digestive, respiratory, circulatory, nervous, the combined eye, ear, face and neck, and other) we assessed this as present vs. absent, where each system has only value per infant. Our end date for infant follow up was January 31, 2022, with mean follow up time of 242.3 days and standard deviation of 104.9 days.

The secondary outcome of birthweight by gestational age was calculated using a published United States birth weight reference (16). Large for gestational age (LGA) was defined as greater than the 90th percentile and small for gestational age (SGA) was defined as less than the 10th percentile of neonatal birth weight. Infants born at less than 24 weeks gestation were placed in their own category as there are no established birth weight curves below this gestational age. Other neonatal outcomes examined include NICU admission and living status.

### Power calculation

Based on estimates from the CDC in conjunction with the MACDP, major structural or genetic birth defects affect approximately 3% of United States births (17). A power calculation was performed after data collection. This study has 80% power using a two-sided chi-square test with a type 1 error of 0.05, to detect a difference in the congenital anomaly rate of 3% (3% vs. 6%) between infants who were and were not exposed to COVID-19 vaccination during the first trimester (at least one dose during first trimester) compared to unvaccinated cohorts.

### Analysis

Comparisons between groups were evaluated using the chi-square test for categorical variables and the Kruskal–Wallis test for continuous variables. A multivariable logistic regression model to assess the association between the outcome of congenital anomalies and vaccination status was adjusted by maternal age at delivery, smoking status, illicit drug use, gravidity, COVID-19 during pregnancy, pre-gestational diabetes, and chronic hypertension. Pre-gestational diabetes was added to the regression model despite being non-significant in the univariate analysis due to the frequency of the condition and its known association with various congenital anomalies (18). Diagnostic codes used for maternal conditions are outlined in Supplementary Table S2.

For neonatal weight at delivery, a nominal regression model was conducted with appropriate for gestational age (AGA) as the reference group and adjusting for the same factors as referenced above. As only 23 infants from 20 patients were less than 24 weeks gestation, this group was removed for modeling purposes. Interactions between COVID-19 infection during pregnancy and COVID-19 vaccination (none, 1st Tri, other) were tested using the likelihood ratio test for both the primary and secondary outcome. All model assumptions were validated (19). Analysis was performed using SAS (version 9.4; SAS Institute Inc., Cary, NC). All calculated *p*-values were 2-sided and any values <0.05 were considered statistically significant.

## Results

**Figure 1:**
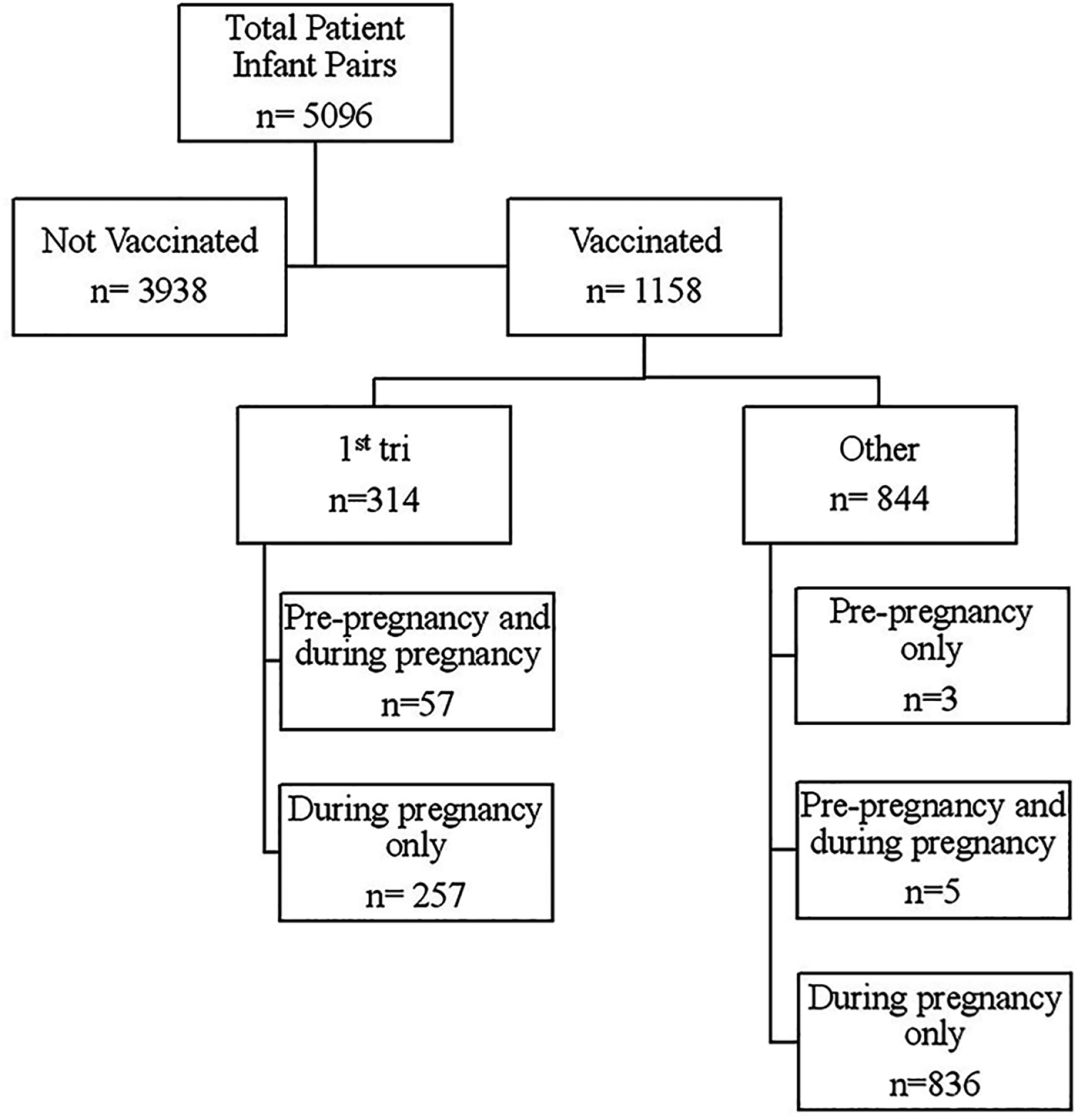
Flow diagram of vaccination timing in relation to pregnancy start. 1st tri = All patients receiving at least one dose of SARS-CoV-2 vaccine in the first trimester. Other = All patients receiving at least one dose of SARS-CoV-2 vaccine as long as no doses were received in the first trimester.

A total of 5,096 patient-infant pairs delivering between December 10, 2020 and December 31, 2021 were included in our analysis. Of these, a total of 3,938 patients were not vaccinated and 1,158 were vaccinated (Figure 1). Of those vaccinated, 314 were vaccinated in the first trimester, with 57 out of those 314 having had at least one dose of the vaccine pre-pregnancy. Of the 844 patients in the other vaccinated group, 3 had doses only prior to pregnancy, 5 had doses prior to and during pregnancy (2nd and 3rd trimesters), and the remaining 836 had doses only in the second and/or third trimesters.

Several patient characteristics differed between vaccinated and unvaccinated groups. Vaccinated pregnant people were significantly older, more likely to be White non-Hispanic, were of lower gravidity and parity, and were more likely to have chronic hypertension. Vaccinated pregnant people were significantly less likely to be obese or have substance use disorder. We found no difference between vaccinated and unvaccinated groups regarding presence of pre-gestational diabetes or multiple pregnancy (Table 1). The rates of gestational diabetes, gestational hypertension, and preeclampsia did not differ significantly between vaccinated and not vaccinated groups. Gestational age at delivery did not differ amongst groups. However, it should be regarded that 6 infants in the unvaccinated group were missing gestational age information. Unvaccinated pregnant people were significantly more likely to have COVID-19 during pregnancy however, the rate of monoclonal antibody (MAB) treatment, the treatment recommended for outpatient COVID-19 illness during the study period, did not differ significantly (Table 1). Among those who were vaccinated and acquired COVID-19 during the pregnancy, most were diagnosed with COVID-19 prior to their first vaccination (Table 2).

**Table 1:** Maternal characteristics of not vaccinated vs. vaccinated patients.

COVID vaccine 2022 Maternal characteristics				
	Not vaccinated (*N* = 3,938)	Vaccinated (*N* = 1,158)	Total (*N* = 5,096)	*P*-value
Maternal age at delivery Mean (SD)	29.1 (5.3)	31.4 (4.3)	29.6 (5.1)	**<0.0001** ^a^
Gravidity mean (SD)	2.7 (1.8)	2.5 (1.7)	2.7 (1.8)	**0.001** ^a^
1	1,073 (27.2%)	347 (30.0%)	1,420 (27.9%)	**0.005** ^b^
2	1,055 (26.8%)	338 (29.2%)	1,393 (27.3%)	
3	771 (19.6%)	225 (19.4%)	996 (19.5%)	
4+	1,039 (26.4%)	248 (21.4%)	1,287 (25.3%)	
Parity mean (SD)	2.2 (1.3)	2.0 (1.1)	2.2 (1.3)	**0.0001** ^a^
1	1,373 (34.9%)	426 (36.8%)	1,799 (35.3%)	**<0.0001** ^b^
2	1,230 (31.2%)	425 (36.7%)	1,655 (32.5%)	
3	749 (19.0%)	207 (17.9%)	956 (18.8%)	
4+	586 (14.9%)	100 (8.6%)	686 (13.5%)	
Race/Ethnicity				**<0.0001** ^b^
Hispanic	383 (9.7%)	64 (5.5%)	447 (8.8%)	
White, non-Hispanic	3,023 (76.8%)	982 (84.8%)	4,005 (78.6%)	
Asian, non-Hispanic	169 (4.3%)	58 (5.0%)	227 (4.5%)	
Black, non-Hispanic	233 (5.9%)	27 (2.3%)	260 (5.1%)	
Other/not disclosed	130 (3.3%)	27 (2.3%)	157 (3.1%)	
Gestational age, (weeks)^c^ Mean (SD)	38.8 (2.1)	38.8 (2.1)	38.8 (2.1)	0.87^a^
37+	3,584 (91.1%)	1,057 (91.3%)	4,641 (91.2%)	0.77^b^
32–36 6/7	300 (7.6%)	83 (7.2%)	383 (7.5%)	
24–31 6/7	33 (0.8%)	13 (1.1%)	46 (0.9%)	
<24	15 (0.4%)	5 (0.4%)	20 (0.4%)	
Obesity (BMI >30)^d^	1,123 (28.5%)	284 (24.5%)	1,407 (27.6%)	**0.01** ^b^
Pre-gestational diabetes	83 (2.1%)	28 (2.4%)	111 (2.2%)	0.52^b^
Gestational diabetes	508 (12.9%)	135 (11.7%)	643 (12.6%)	0.26^b^
Chronic HTN	217 (5.5%)	85 (7.3%)	302 (5.9%)	**0.02** ^b^
Pre-eclampsia/gestational hypertension	528 (13.4%)	174 (15.0%)	702 (13.8%)	0.16^b^
Substance use	127 (3.2%)	20 (1.7%)	147 (2.9%)	0.007^b^
COVID-19 during pregnancy	507 (12.9%)	86 (7.4%)	593 (11.6%)	**<0.0001** ^b^
MAB^e^ treatment	24 (0.6%)	6 (0.5%)	30 (0.6%)	0.72^b^
Multiples				0.89^b^
Singleton	3,879 (98.5%)	1,140 (98.4%)	5,019 (98.5%)	
Twins (all types)	59 (1.5%)	18 (1.6%)	77 (1.5%)	
Monochorionic diamniotic gestations	11/59 (18.6%)	5/18 (27.8%)	16/77 (20.8%)	0.40^b^

Bold indicates statistical significance.

^a^ Kruskal–Wallis.

^b^ Chi-square.

^c^ Missing 6 values in the not vaccinated group.

^d^ Missing 746 observations (621 in the not vaccinated cohort).

^e^ MAB, monoclonal antibody treatment.

**Table 2:** Timing of COVID-19 in relation to vaccination timing.

COVID-19 prior to any vaccine	COVID-19 after dose 1 but prior to dose 2	COVID-19 after dose 2	*N* (%)
		Yes	30 (35%)
	Yes		7 (8%)
Yes			49 (57%)

No significant difference in the composite congenital anomalies outcome was observed when examining infant characteristics by not vaccinated, vaccinated, or 1st trimester vaccination groups (Table 3, 20.0% vs. 19.1% vs. 19.1%, 1st Trimester *p*-value 0.72, Vaccinated *p*-value 0.53). When further examining congenital anomalies by organ system, we did demonstrate a significant difference in eye, ear, face, neck anomalies between vaccinated and not vaccinated groups (Table 3, Not vaccinated = 2.3%, Vaccinated = 3.3%, *p*-value 0.04), but we did not demonstrate this difference between the 1st trimester and not vaccinated groups (Table 3, Not vaccinated = 2.3%, 1st Trimester = 2.5%, *p*-value 0.77). The relative risk for an ENT anomaly among vaccinated infants was 1.47 times more likely than unvaccinated infants, with the 95% confidence interval ranging from 1.02 to 2.13 times. No differences were found between not vaccinated, vaccinated, or 1st trimester vaccinated groups for any other organ systems. We manually reviewed the specific eye, ear, face, neck anomalies diagnosed in not vaccinated vs. vaccinated groups (Table 4). None of these anomalies would be considered major congenital anomalies, with all requiring either no intervention or minor surgical procedures (15). We found no difference for infant sex, NICU admission, and living status across all groups. When adjusting for covariates listed in the statistical section, no significant difference was found by vaccination status (Table 5, 1st Trimester Vaccine OR = 0.92 95% CI 0.69–1.24, Other Vaccine OR = 0.93 95% CI 0.77–1.13).

**Table 3:** Infant characteristics by COVID-19 vaccination status.

COVID vaccine 2022 Infant characteristics					
	Not vaccinated (*N* = 3,997)	Vaccinated (*N* = 1,176)	Any vaccine in the 1st trimester (*N* = 319)	*P*-value any vaccine 1st tri vs. not vaccinated	*P*-value vaccinated vs. not vaccinated
Infant sex				0.21^a^	0.75^a^
Female	1,946 (48.7%)	587 (49.9%)	163 (51.1%)		
Male	2,043 (51.1%)	587 (49.9%)	154 (48.3%)		
Unknown	8 (0.2%)	2 (0.2%)	2 (0.6%)		
Birthweight (g): mean (SD)	3,336.9 (594.8)	3,320.7 (603.3)	3,302.9 (674.9)	0.70^b^	0.59^b^
Birthweight categories^c,e^				0.22^a^	0.99^a^
SGA	284 (7.1%)	87 (7.4%)	19 (6.0%)		
AGA	3,170 (79.3%)	933 (79.3%)	252 (79.0%)		
LGA	518 (13.0%)	150 (12.8%)	43 (13.5%)		
GA <24 weeks	18 (0.5%)	5 (0.4%)	5 (1.6%)		
Congenital anomalies^d^					
Composite	798 (20.0%)	225 (19.1%)	61 (19.1%)	0.72^a^	0.53^a^
Chromosomal abnormalities	16 (0.4%)	4 (0.3%)	2 (0.6%)	0.55^a^	0.77^a^
Musculoskeletal system	173 (4.3%)	52 (4.4%)	15 (4.7%)	0.75^a^	0.89^a^
Urinary system	58 (1.5%)	21 (1.8%)	4 (1.3%)	0.78^a^	0.41^a^
Genital organs	170 (4.3%)	39 (3.3%)	9 (2.8%)	0.22^a^	0.15^a^
Digestive system	19 (0.5%)	8 (0.7%)	2 (0.6%)	0.71^a^	0.39^a^
Respiratory system	69 (1.7%)	16 (1.4%)	5 (1.6%)	0.83^a^	0.389^a^
Circulatory system	185 (4.6%)	56 (4.8%)	21 (6.6%)	0.12^a^	0.85^a^
Nervous system	33 (0.8%)	8 (0.7%)	3 (0.9%)	0.83^a^	0.62^a^
Eye, ear, face, neck	90 (2.3%)	39 (3.3%)	8 (2.5%)	0.77^a^	**0.04** ^a^
Other	198 (5.0%)	43 (3.7%)	11 (3.4%)	0.23^a^	0.68^a^
NICU admission	300 (7.5%)	98 (8.3%)	27 (8.5%)	0.53^a^	0.35^a^
Living status				0.15^a^	0.68^a^
Stillbirth	11 (0.3%)	2 (0.2%)	1 (0.3%)		
Living	3,975 (99.4%)	1,171 (99.6%)	316 (99.1%)		
Neonatal demise	10 (0.3%)	2 (0.2%)	1 (0.3%)		
Therapeutic abortion	1 (0.0%)	1 (0.1%)	1 (0.3%)		

Bold indicates statistical significance.

^a^ Chi-square.

^b^ Kruskal–Wallis.

^c^ SGA, small for gestational age; LGA, large for gestational age; AGA, appropriate for gestational age; GA, gestational age.

^d^ At least one anomaly present.

^e^ Seven missing gestational age, all in unvaccinated group.

**Table 4:** Eye, ear, face, neck anomalies by vaccination status.

Eye, ear, face, neck system diagnosis	ICD-10 code	Charted anomaly	Not vaccinated (*N* = 3,997)	Vaccinated (*N* = 1,176)
Accessory auricle	Q17.0		25 (0.63%)	3 (0.26%)
Congenital cataract	Q12.0		1 (0.03%)	0 (0%)
Coloboma of iris	Q13.0		1 (0.03%)	0 (0%)
Coloboma of lens	Q12.2		0 (0%)	2 (0.17%)
Congenital corneal opacity	Q13.3		0 (0%)	1 (0.09%)
Congenital malformation of ear, unspecified	Q17.9	Bilateral helical rim folding	2 (0.05%)	3 (0.26%)
		Bilateral underdevelopment of helical rim	2 (0.05%)	0 (0%)
		Bilateral helical rim flattening	1 (0.03%)	2 (0.17%)
		Bilateral cupped ears	1 (0.03%)	1 (0.09%)
		Left helical rim folding	1 (0.03%)	0 (0%)
		Left tragus skin tag	0 (0%)	1 (0.09%)
		Left ear conchal bowl deformity	1 (0.03%)	0 (0%)
		Bilateral folding of scaphoid fossa	0 (0%)	1 (0.09%)
		Right lop deformity	0 (0%)	1 (0.09%)
		Left earlobe cleft	1 (0.03%)	0 (0%)
		CHARGE syndrome^a^	0 (0%)	1 (0.09%)
Congenital malformations of face and neck, unspecified	Q18.9	Bilateral nasal lacrimal duct cysts	1 (0.03%)	0 (0%)
		Laryngomalacia	1 (0.03%)	0 (0%)
Congenital ptosis	Q10.0		1 (0.03%)	1 (0.09%)
Congenital stenosis and stricture of lacrimal duct	Q10.5		35 (0.88%)	11 (0.94%)
Cyst branchial cleft	Q18.0		0 (0%)	1 (0.09%)
Microtia	Q17.2		0 (0%)	1 (0.09%)
Misplaced ear	Q17.4		2 (0.05%)	0 (0%)
Obstruction nasolacrimal duct congenital	Q10.5		2 (0.05%)	0 (0%)
Other branchial cleft malformations	Q18.2	Cervical chondrocutaneous branchial remnant	0 (0%)	1 (0.09%)
Other misshapen ear	Q17.3	Bilateral lop ear	1 (0.03%)	0 (0%)
		Bilateral cupped ear	0 (0%)	1 (0.09%)
		Bilateral helical rim folding	0 (0%)	1 (0.09%)
Other specified congenital malformations of ear	Q17.8	Right pinna with exaggerated ear folding	1 (0.03%)	0 (0%)
		Bilateral dysplastic ears^b^	0 (0%)	1 (0.09%)
Preauricular sinus and cyst	Q18.1		7 (0.18%)	3 (0.26%)
Prominent ear	Q17.5		1 (0.03%)	0 (0%)
Tag skin ear	Q17.0		2 (0.05%)	1 (0.09%)
Webbing of neck	Q18.3		0 (0%)	1 (0.09%)

Eye, ear, face, neck, system diagnosis is the name of the ICD-10 diagnostic code under which the anomaly was labeled. If the code used was deemed to be non-specific, the chart was then manually reviewed. The charted anomaly by the provider team was then noted.

^a^ Neonate died at 6 months of life due to complications of CHARGE syndrome.

^b^ Neonate died at 6 months of life due to myotonic dystrophy.

**Table 5:** Multivariate model of infant characteristics by COVID-19 vaccination status.

Outcome	Vaccination status	Adjusted odds ratio (95% CI)	*P*-value^b^
Congenital anomaly—Composite^a^ (Ref: none)	Not vaccinated	Reference	0.68
	1st trimester vaccine	0.92 (0.69, 1.24)	
	Other vaccine	0.93 (0.77, 1.13)	
Birthweight^c^ (Ref: AGA)			
SGA	Not vaccinated	Reference	0.79
	1st trimester vaccine	0.89 (0.54, 1.44)	
	Vaccinated other	1.14 (0.86, 1.52)	
LGA	Not vaccinated	Reference	
	1st Trimester vaccine	1.01 (0.72, 1.43)	
	Vaccinated other	0.94 (0.75, 1.18)	

Adjusted by COVID-19 status during pregnancy, age at delivery, smoking status, illicit drug use, gravidity, presence of pre-gestational diabetes, and presence of chronic hypertension.

1st trimester vaccine = All patients receiving at least one dose of SARS-CoV-2 vaccine in the first trimester.

Other vaccine = All patients receiving at least one dose of SARS-CoV-2 vaccine as long as no doses were received in the first trimester.

SGA, small for gestational age; LGA, large for gestational age.

^a^ Infants with multiple congenital anomalies counted once in the composite.

^b^ Wald Chi Square test statistic.

^c^ Patients with GA <24 weeks omitted from the model due to small sample size.

**Figure 2:**
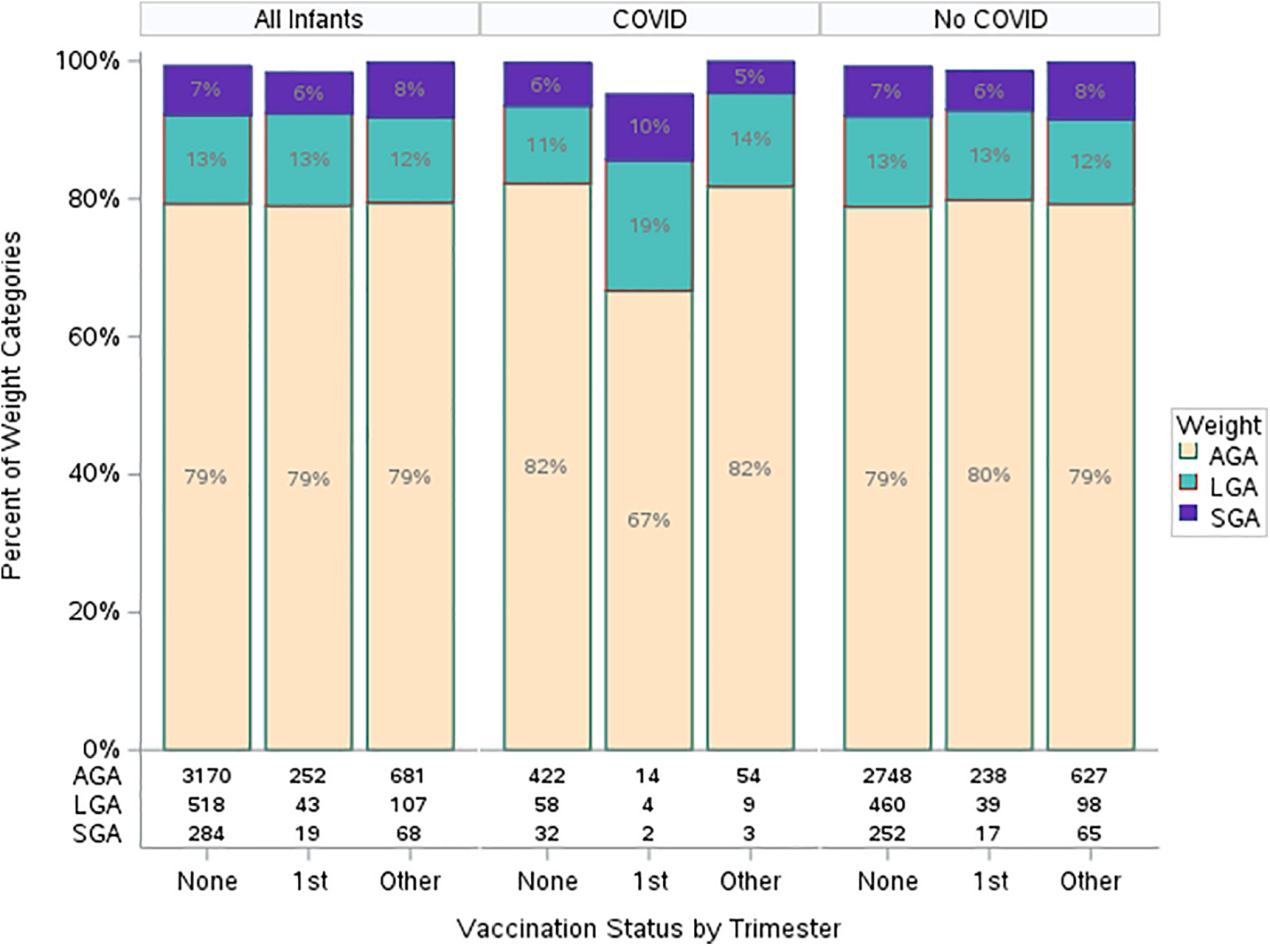
Gestational weight by vaccination and COVID-19 infection status. 1st trimester vaccine = All patients receiving at least one dose of SARS-CoV-2 vaccine in the first trimester. Other vaccine = All patients receiving at least one dose of SARS-CoV-2 vaccine as long as no doses were received in the first trimester. SGA, small for gestational age; LGA, large for gestational age; AGA, appropriate for gestational age; GA, gestational age.

Lastly, we examined the association of birthweight with vaccination status. No difference in birthweight was observed when analyzed by vaccination status in any trimester or vaccination in first trimester (Table 3). Of note, there were 7 missing gestational ages in the unvaccinated group and so these could not be analyzed. No association was found when adjusting by covariates within a nominal multivariable model (Table 5). Interaction between birthweight by gestational age, vaccine status, and COVID-19 infection was found not to be statistically significant (Figure 2).

## Comment

### Principal findings

In this prospective cohort study, COVID-19 vaccination status including vaccination during the first trimester was not associated with an increased risk of composite congenital anomalies. We did notice a significant difference in the presence of minor eye, ear, face, neck anomalies among vaccinated vs. not vaccinated groups (Table 3, Not vaccinated = 2.3%, Vaccinated = 3.3%, *p*-value 0.04). However, we did not demonstrate this difference between 1st trimester vaccination and not vaccinated groups (Table 3, Not vaccinated = 2.3%, 1st Trimester = 2.5%, *p*-value 0.77). No other associations were found between not vaccinated, vaccinated, and first trimester vaccination groups for anomalies by any other organ systems. We did not see differences in birthweight by gestational age, APGAR scores, incidence of NICU admission, or living status of the neonate. COVID-19 vaccination status in pregnancy was not associated with increased rates of pregnancy complications including gestational diabetes, gestational hypertension, and preeclampsia.

### Results in the context of what is known

COVID-19 vaccination in pregnancy has been shown to be similarly effective as vaccination in the non-pregnant population, with one study demonstrating 89% (43%–100%) prevention of COVID-19-related hospitalizations in pregnant populations (20) and another demonstrating risk of progression to severe disease was reduced by 74% after the primary series and 91% after the booster dose (21). Given the known increased risk of mortality and severe COVID-19 disease in pregnant people, there is a clear patient benefit for vaccination (1, 2, 13). Additionally, data has shown vaccination is associated with improved pregnancy outcomes and may be beneficial to offspring. Several studies demonstrate associated reduced risk of stillbirth, neonatal death, and premature delivery with vaccination administration during pregnancy (22–24). One meta-analysis demonstrated significantly lower risk of stillbirth by 15% in vaccinated cohorts (25). Evidence has also accrued regarding vaccine efficacy in infants after vaccination administration during pregnancy. The presence of functional anti-spike IgG antibodies in umbilical cord blood has been shown (26, 27), with one study demonstrating 61% (95% CI = 31%–78%) effectiveness of COVID-19 vaccination during pregnancy against critical illness and hospitalization from COVID-19 infection among infants aged <6 months (28, 29). Infant COVID-19 infection and hospitalization prevention associated with maternal vaccination during pregnancy has now been corroborated in Canadian, Israeli, and Norwegian population studies (30–32).

Many trials have now examined neonatal outcomes as related to COVID-19 vaccination in pregnancy (22, 33–39). One large retrospective cohort study following 46,079 pregnant people demonstrated no increased risk of small for gestational age infants; however, only 1.7% of the study population received the vaccine in the first trimester (37). A large Swedish cohort of 94,303 neonates exposed to the vaccine during pregnancy exhibited no increased risk of adverse neonatal outcomes including complications such as disorders of the nervous, circulatory, respiratory and gastrointestinal systems as well as hematologic and infectious complications (40). Several studies have also emerged surrounding the safety of vaccination as it pertains to congenital anomalies. Another study examining the presence of congenital anomalies at time of anatomy ultrasound did not find a difference associated with vaccination. Remarkably, 1,149 (43.8%) of those vaccinated did receive the vaccine during the teratogenic window, however, this study remains limited in that it did not examine congenital anomalies as diagnosed in neonatal life (38). A population registry of all singleton livebirths in Israel demonstrated no association between neonatal anomalies and vaccine uptake during pregnancy, but by design excluded fetuses who miscarried or were terminated (36). Perhaps the most robust study to date on the subject was conducted by Calvert et al. This was a matched cohort population study in Scotland finding no association between vaccination 6 weeks pre-conception to 19 weeks gestational age and major congenital anomalies diagnosed in neonatal life (35). This study included all clinically recognized pregnancies ending in any outcome.

### Clinical implications

Evidence is rapidly accruing regarding the safety of COVID-19 vaccination for pregnant people and their offspring. Our study adds to this data and examines the association of vaccination in pregnancy and congenital anomalies diagnosed in neonatal life, with no increased risk of composite congenital anomalies. We did demonstrate a significant difference in eye, ear, face, neck anomalies between vaccinated and not vaccinated groups but did not demonstrate this difference between the first trimester and not vaccinated groups. It seems unlikely that vaccination itself would be the cause of the increase in ENT anomalies seen as this increased risk was not observed after vaccination in the first trimester when the fetus would be at highest teratogenic risk. It may be possible that there are other differences between populations vaccinated in first vs. later trimesters that could account for this difference. This finding may also be a type 1 error in our study. Calvert et al. does examine the presence of eye, ear, face, neck anomalies, however, their analysis is limited to major anomalies while our analysis includes all anomalies. Table 4 outlines the eye, ear, face, neck anomalies found in our study, with most being considered minor and/or cosmetic in nature, requiring no or minimal interventions (35). Based off the amounting data from other studies, we would encourage patients to pursue vaccination in all trimesters of pregnancy. In addition, our study agrees with previous studies on the lack of association between COVID-19 vaccination in pregnancy and small for gestational age neonates (22, 23, 34, 37). These results can be used to counsel pregnant patients making decisions regarding vaccination.

### Research implications

While our results are reassuring, a minority (*n* = 314, 27.1%) of our vaccinated cohort was vaccinated in the first trimester and additional studies will be needed to examine differences in rare adverse birth outcomes following early pregnancy vaccination. Further research needs to be conducted specifically examining the association of neonatal outcomes with first trimester vaccination—the time of organogenesis—to better define risks of COVID vaccination. In addition, future studies should include a diverse population at the multicenter level as our study was limited demographically. Lastly, our study was conducted prior to the introduction of the bivalent COVID-19 vaccine. Further studies are needed examining this formulation as this is the predominant COVID-19 vaccine given today.

### Strengths and limitations

The strengths of this study include the use of a comprehensive population level vaccine registry data in combination with a validated, all-inclusive delivery database including births at multiple community and teaching hospitals across two states. Data were extracted from the primary medical record, and all identified congenital anomalies were verified by medical record review. Limitations of this analysis include the small percentage of non-White subjects in taken from a small geographic region which may not be representative of a more diverse patient population. Additionally, a minority (*n* = 320, 20.1%) of our vaccinated cohort was vaccinated in the first trimester. We had 80% power to detect a difference between unvaccinated and first trimester vaccine cohorts at a 3% difference (3% vs. 6%). It is possible that a smaller difference exists that was unable to be detected. Although the incidence of chromosomal anomalies and monochorionic-diamniotic gestations appear to be similar in both groups, we did not control for this in our analysis. We did examine teratogenicity as a cause of congenital anomalies (ICD-10 code Q86.xx), however, this was also not controlled for in our analysis. Substance use was most common in the unvaccinated group, but we cannot report on the specific use of teratogenic substance or medications beyond this. Finally, this study was conducted prior to the advent of the bivalent version of the SARS-CoV-2 vaccine, protecting against the Omicron BA.4/BA.5 variant, the principal vaccine given today.

### Conclusions

Our findings add to the existing research regarding the safety of COVID-19 vaccination as it pertains to pregnancy and neonatal outcomes. We corroborate prior evidence that COVID-19 vaccination is not associated with adverse pregnancy outcomes or increased incidence of small for gestational age neonates. With this study, we add new information regarding the safety of COVID-19 vaccination as it pertains to composite neonatal congenital anomalies, with no association demonstrated. This data should encourage patients and providers to pursue vaccination in all trimesters of pregnancy.

## Data Availability

These findings were presented at the SMFM 43rd Annual Pregnancy Meeting in San Francisco, CA. February 9, 2023.

## Data availability statement

The data analyzed in this study is subject to the following licenses/restrictions: protected electronic health record information. Requests to access these datasets should be directed to theiler.regan@mayo.edu.

## Appendix A Vaccination dose timing in relation to pregnancy start.

**Table:**

Vaccine group	Dose 1	Dose 2	Dose 3	*N* (%)
1st	Less than equal to 30 days pre-pregnancy	Less than equal to 30 days pre-pregnancy	1st trimester	1 (0.1%)
1st	Less than equal to 30 days pre-pregnancy	1st trimester		28 (2.4%)
1st	Less than equal to 30 days pre-pregnancy	1st trimester	3rd trimester	28 (2.4%)
1st	1st trimester			19 (1.6%)
1st	1st trimester	1st trimester		125 (10.8%)
1st	1st trimester	1st trimester	3rd trimester	30 (2.6%)
1st	1st trimester	2nd trimester		76 (6.6%)
1st	1st trimester	2nd trimester	3rd trimester	1 (0.1%)
1st	1st trimester	3rd trimester		6 (0.5%)
Other	Less than equal to 30 days pre-pregnancy	Less than equal to 30 days pre-pregnancy		3 (0.3%)
Other	Less than equal to 30 days pre-pregnancy	Less than equal to 30 days pre-pregnancy	2nd trimester	1 (0.1%)
Other	Less than equal to 30 days pre-pregnancy	Less than equal to 30 days pre-pregnancy	3rd trimester	3 (0.3%)
Other	Less than equal to 30 days pre-pregnancy	3rd Trimester		1 (0.1%)
Other	2nd trimester			25 (2.2%)
Other	2nd trimester	2nd trimester		336 (29%)
Other	2nd trimester	2nd trimester	2nd trimester	1 (0.1%)
Other	2nd trimester	3rd trimester		110 (9.5%)
Other	3rd trimester			97 (8.4%)
Other	3rd trimester	3rd trimester		267 (23.1%)

1^st^: All patients receiving at least one dose of SARS-CoV-2 vaccine in the first trimester.

Other: All patients receiving at least one dose of SARS-CoV-2 vaccine as long as no doses were received in the first trimester.

## Appendix B Vaccine manufacturers by dose.

**Table:**

Dose 1 manufacturer	Dose 2 manufacturer	Dose 3 manufacturer	*N* (%)
Unknown			134 (11.57%)
Janssen^a^			21 (1.81%)
Janssen^a^	Janssen^a^		4 (0.34%)
Janssen^a^	Moderna US, Inc.		3 (0.26%)
Janssen^a^	Pfizer, Inc.		9 (0.78%)
Janssen^a^	Pfizer, Inc.	Pfizer, Inc.	1 (0.09%)
Merck & Co. Inc.^b,d^	Moderna US, Inc.	Pfizer, Inc.	1 (0.09%)
Moderna US, Inc.			8 (0.69%)
Moderna US, Inc.	Moderna US, Inc.		116 (10.02%)
Moderna US, Inc.	Moderna US, Inc.	Moderna US, Inc.	95 (8.20%)
Moderna US, Inc.	Moderna US, Inc.	Pfizer, Inc.	16 (1.38%)
Moderna US, Inc.	Pfizer, Inc.	Pfizer, Inc.	1 (0.09%)
Pfizer, Inc.			14 (1.21%)
Pfizer, Inc.	Pfizer, Inc.		283 (24.44%)
Pfizer, Inc.	Pfizer, Inc.	Moderna US, Inc.	9 (0.77%)
Pfizer, Inc.	Pfizer, Inc.	Pfizer, Inc.	442 (38.17%)
Pfizer, Inc.	Pfizer, Inc.	Sanofi Pasteur^c^	1 (0.09%)

^a^ Janssen: viral vector vaccine containing the gene encoding the spike protein of SARS-CoV-2.

Moderna US, Inc.: nucleoside-modified mRNA encoding the spike protein of SARS-CoV-2.

Pfizer, Inc.: nucleoside-modified mRNA encoding the spike protein of SARS-CoV-2.

^b^ Merck & Co. Inc.: V590: live recombinant viral vector vaccine based on vesicular stomatitis virus.

V591: live recombinant viral vector vaccine based on the measles virus.

^c^ Sanofi Pasteur: recombinant protein subunit vaccine containing the spike protein of SARS-CoV-2.

^d^ Unknown if received V590 or V591 Merck and Co. Inc. vaccine.
