# Supplementary material for: Maternal COVID-19 vaccination status and association with neonatal congenital anomalies
Source: Front Pediatr. 2024 Apr 19;12:1355502. doi: 10.3389/fped.2024.1355502 (PMC11066299; doi:10.3389/fped.2024.1355502)
Supplement: Supplementary file 1 [file Datasheet1.pdf]

Supplemental Table 1: Diagnosis Codes for Congenital Anomalies

**Congenital Malformations Description**	**ICD-10 Code**
Nervous System	Q00.xx, Q01.xx, Q02.xx, Q03.xx, Q04.xx, Q05.xx, Q06.xx, Q07.xx
Eye, Ear, Face, Neck	Q10.xx, Q11.xx, Q12.xx, Q13.xx, Q14.xx, Q15.xx, Q16.xx, Q17.xx, Q18.xx
Circulatory System	Q20.xx, Q21.xx, Q22.xx, Q23.xx, Q24.xx, Q25.xx, Q26.xx, Q27.xx, Q28.xx
Respiratory System	Q30.xx, Q31.xx, Q32.xx, Q33.xx, Q34.xx
Cleft Lip and/or Palate	Q35.xx, Q36.xx, Q37.xx
Digestive System	Q38.xx, Q39.xx, Q40.xx, Q41.xx, Q42.xx, Q43.xx, Q44.xx, Q45.xx
Genital Organs	Q50.xx, Q51.xx, Q52.xx, Q53.xx, Q54.xx, Q55.xx, Q56.xx
Chromosomal Abnormalities	Q90.xx, Q91.xx, Q92.xx, Q93.xx, Q95.xx, Q96.xx, Q97.xx, Q98.xx, Q99.xx
Urinary System	Q60.xx, Q61.xx, Q62.xx, Q63.xx, Q64.xx
Musculoskeletal System	Q65.xx, Q66.xx, Q67.xx, Q68.xx, Q69.xx, Q70.xx, Q71.xx, Q72.xx, Q73.xx, Q74.xx, Q75.xx, Q76.xx, Q77.xx, Q78.xx, Q79.xx<br>Except Q67.3
Other	Q80.xx, Q81.xx, Q82.xx, Q83.xx, Q84.xx, Q85.xx, Q86.xx, Q87.xx, Q89.xx<br>Except Q82.6, Q82.5

Supplemental Table 2: Diagnosis Codes for Maternal Conditions

**Diagnosis Description**	**ICD-10 Code**
Gestational Diabetes	O24.4
Gestational Hypertension	O13.xx
Chronic Hypertension	O10.xx
Pre Gestational Diabetes	O24.01, O24.011, O24.012, O24.013, O24.019, O24.02, O24.03, O24.111, O24.112, O24.113, O24.119, O24.12, O24.13, O24.311, O24.312, O24.313, O24.319, O24.32, O24.33
Pre-Eclampsia	O11.xx
Obesity	Pre-pregnancy BMI > 30
